# Temporal factors in the extinction of fear in inbred mouse strains differing in extinction efficacy

**DOI:** 10.1186/2045-5380-3-13

**Published:** 2013-07-05

**Authors:** Kathryn MacPherson, Nigel Whittle, Marguerite Camp, Ozge Gunduz-Cinar, Nicolas Singewald, Andrew Holmes

**Affiliations:** 1 Laboratory of Behavioral and Genomic Neuroscience, National Institute on Alcohol Abuse and Alcoholism, NIH, Bethesda, MD, USA; 2 Department of Pharmacology & Toxicology, Institute of Pharmacy and CMBI, University of Innsbruck, Innsbruck, Austria

**Keywords:** Mouse, Gene, Behavior, Fear, Second order conditioning, PTSD, Prefrontal cortex, Amygdala, Anxiety, Rodent, Exposure-based therapy

## Abstract

**Background:**

Various neuropsychiatric conditions, including posttraumatic stress disorder (PTSD), are characterized by deficient fear extinction, but individuals differ greatly in risk for these. While there is growing evidence that fear extinction is influenced by certain procedural variables, it is unclear how these influences might vary across individuals and subpopulations. To model individual differences in fear extinction, prior studies identified a strain of inbred mouse, 129S1/SvImJ (S1), which exhibits a profound deficit in fear extinction, as compared to other inbred strains, such as C57BL/6J (B6).

**Methods:**

Here, we assessed the effects of procedural variables on the impaired extinction phenotype of the S1 strain and, by comparison, the extinction-intact B6 strain. The variables studied were 1) the interval between conditioning and extinction, 2) the interval between cues during extinction training, 3) single-cue exposure before extinction training, and 4) extinction of a second-order conditioned cue.

**Results:**

Conducting extinction training soon after (‘immediately’) conditioning attenuated fear retrieval in S1 mice and impaired extinction in B6 mice. Spacing cue presentations with long inter-trial intervals during extinction training augmented fear in S1 and B6 mice. The effect of spacing was lost with one-trial fear conditioning in B6, but not S1 mice. A single exposure to a conditioned cue before extinction training did not alter extinction retrieval, either in B6 or S1 mice. Both the S1 and B6 strains exhibited robust second-order fear conditioning, in which a cue associated with footshock was sufficient to serve as a conditioned exciter to condition a fear association to a second cue. B6 mice extinguished the fear response to the second-order conditioned cue, but S1 mice failed to do so.

**Conclusions:**

These data provide further evidence that fear extinction is strongly influenced by multiple procedural variables and is so in a highly strain-dependent manner. This suggests that the efficacy of extinction-based behavioral interventions, such as exposure therapy, for trauma-related anxiety disorders will be determined by the procedural parameters employed and the degree to which the patient can extinguish.

## Background

Fear extinction is a form of learning in which a conditioned fear response is reduced by repeated exposure to the conditioned stimulus (CS) in the absence of the unconditioned stimulus (US) [1]. Various neuropsychiatric conditions, including posttraumatic stress disorder (PTSD) [2] and schizophrenia [3], are characterized by deficient fear extinction. Measuring fear extinction in rodents has emerged as a valuable translational assay for studying both the neural basis of poor extinction [4,5], and identifying novel therapeutic approaches that facilitate extinction [6-8].

Individuals differ greatly in risk for anxiety disorders, including PTSD, in part likely due to the moderating influence of genetic factors [9,10]. With this in mind, recent studies have sought to identify neural mechanisms underlying differences in fear and extinction behaviors between subgroups selected from larger populations [11-13], or identify inbred (isogenic) rodent strains that show reliable trait differences in fear extinction [14]. In this context, we recently identified an inbred strain of mouse, 129S1/SvImJ (S1), that exhibits a profound deficit in fear extinction, as compared to a reference inbred strain, C57BL/6J (B6) [15-19].

Supporting the validity of the S1 inbred mouse strain as a model for the impairment of extinction seen in anxiety disorders, the phenotypic abnormalities in these mice extend to a range of behavioral, autonomic and neuroendocrine disturbances, some of which are characteristic of anxiety disorders including PTSD [18]. Moreover, deficient extinction in these mice can be rescued by chronic treatment with fluoxetine [18], a medication for PTSD and other anxiety disorders, and also by various putative novel therapeutics, including an endocannabinoid-boosting drug [20], histone deacetylase inhibition, and deep brain stimulation [19]. At the neural level, impaired extinction in S1 mice is associated with dendritic hypertrophy in basolateral amygdala (BLA) neurons and a failure to properly recruit corticoamygdala regions mediating extinction [15,17,18]. Importantly, this circuitry can be effectively re-engaged by manipulations that normalize fear extinction in parallel (e.g., dietary zinc restriction) [17]. Taken together, these previous findings suggests that the S1 mouse strain may be a tractable model for studying of the underlying neurobiology and genetics of extinction, and also as a screen for identifying novel interventions that rescue impairments in fear extinction.

There is growing evidence that the efficacy of fear extinction is strongly influenced by the interval between extinction training and the conditioning event and the order and timing of cue presentations during extinction training [21,22]. Such procedural variables could potentially be important considerations when applying extinction-based procedures to clinical settings, in the form of exposure therapy. However, the influence of these variables has not been well studied in the models of impaired extinction despite the fact that the clinical application of exposure therapy typically occurs in extinction impaired patient populations. Therefore, the main aim of the current study was to assess the effects of certain procedural variables on the impaired extinction phenotype of the S1 mouse strain and, by comparison, the good extinguishing B6 mouse strain. The specific variables we studied were 1) the interval between conditioning and extinction [23-25], 2) the interval between cues during extinction training [26-28], 3) single-cue exposure before extinction training [29-32], and 4) extinction of a second-order conditioned cue [33,34].

## Methods

### Subjects

Subjects were male S1 and B6 mice obtained at ~8-9 weeks of age from The Jackson Laboratory (Bar Harbor, ME) (for experiments at NIAAA) or Charles River (Sulzfeld, Germany) (for experiments at University of Innsbruck). Mice were housed 2–4 per cage in a temperature (~22°C) and humidity (NIAAA = 45 ± 15%, University of Innsbruck = 50-60%) controlled *vivarium* under a 12 hour light/dark cycle (NIAAA = lights on 0600 h, University of Innsbruck = lights on 0700 h). The number of mice used in each experiment is given in the figure legends. Experiments were conducted at the NIAAA with the exception of the second-order conditioning experiment, which was conducted at the University of Innsbruck. All procedures were approved by the NIAAA Animal Care and Use Committee or the Austrian Animal Experimentation Ethics Board (Bundesministerium für Wissenschaft und Verkehr, Kommission für Tierversuchsangelegenheiten), and followed the NIH guidelines outlined in ‘Using Animals in Intramural Research.’

### General procedures for fear conditioning and extinction

Unless specified differently below, testing consisted of 3 phases: conditioning, extinction training and extinction retrieval. Freezing (no visible movement except that required for breathing) was manually scored every 5 seconds as an index of fear [35], and converted to a percentage [(number of freezing observations/total number of observations) × 100].

**Conditioning** was conducted in Context A: 27 × 27 × 11 cm (NIAAA) or 25 × 25 × 35 cm (University of Innsbruck) chamber with transparent walls and a metal rod floor, cleaned with a 79.5% water/19.5% ethanol/1% vanilla-extract solution (NIAAA) or water (University of Innsbruck). After a 180-seconds (NIAAA) or 120-second (University of Innsbruck) baseline period, mice received 3 × pairing(s) of a 30-second, 75–80 dB, white noise (NIAAA) or 75 dB 10 kHz pure tone (used as CS1 in second-order conditioning experiments at the University of Innsbruck) conditioned stimulus (CS) and 2 sec, 0.6 mA scrambled footshock [unconditioned stimulus (US)], in which the US was presented during the last 2 seconds of the CS and the inter-trial-interval (ITI) was variable. There was a 120-second no-stimulus period after the final pairing before mice were returned to the home cage.

**Extinction training** was conducted the day after conditioning, in Context B. At NIAAA, this was a 20 cm-diameter Plexiglas cylinder with black/white-checkered walls, solid-Plexiglas opaque floor, cleaned with a 99% water/ 1% acetic acid solution, located in a different room from Context A. At the University of Innsbruck, Context B was a 25 × 25 × 35 cm cage with a solid grey floor and black walls, cleaned with 100% ethanol. CS presentations began after a 180-second (NIAAA) or 120-second (University of Innsbruck) baseline period. Extinction training data were calculated in 5 × CS trial-blocks. Data were analyzed using the first and last extinction trial-blocks in order to allow for consistent comparison across experiments because fewer than 50 CS presentations were given during extinction training in some experiments (i.e., 10 × CS massed and 10 × CS spaced presentations in the massed vs. spaced experiment).

The following day, **extinction retrieval** was tested in Context B. After a 180-second baseline period, mice received 3 × 30-second non-reinforced CS presentations (5 second inter-stimulus interval). Stimulus presentation was controlled by the Med Associates VideoFreeze system (Med Associates, Burlington, VT, USA) (NIAAA) or a TSE operant system (TSE, Bad Homburg, Germany) (University of Innsbruck).

### Effects of varying the interval between conditioning and extinction training

**Figure 1:**
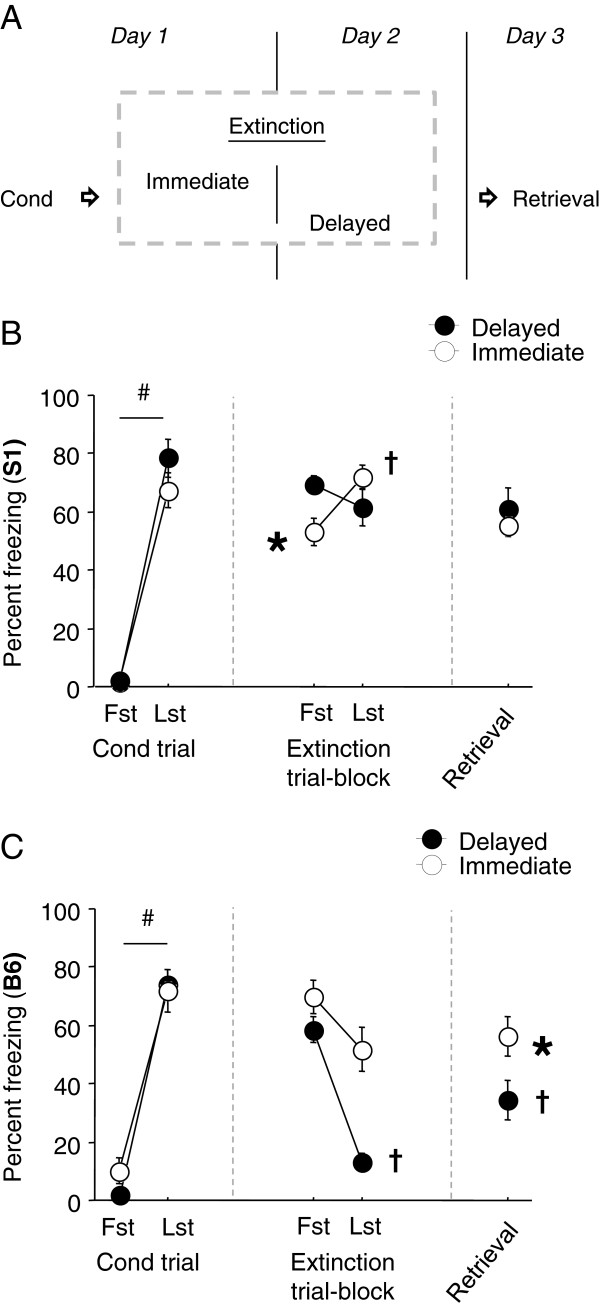
**Effects of immediate extinction training. (A)** Mice received extinction training 10 minutes (immediate) or 1 day (delayed) after fear conditioning. All mice then received equivalent extinction training and retrieval testing. **(B)** In S1 mice, freezing was significantly lower in the immediate than the delayed group during the first extinction trial-block, and then increased across trial-blocks in the immediate but not delayed group. Freezing was similar between groups during extinction retrieval and neither group showed a decrease in freezing on retrieval as compared to the first extinction trial-block (n = 13-19). **(C)** In B6 mice, freezing significantly decreased across extinction trial-blocks in the delayed but only showed a non-significant trend for a decrease in the immediate group. Freezing was significantly higher in the immediate than delayed group during extinction retrieval, although both groups froze less on retrieval than the first trial-block of extinction training (n = 10). Data are means ± SEM. #*P* < .05 last (Lst) vs. first (Fst) conditioning trial, †*P* < .05 Lst extinction trial-block or retrieval vs. Fst extinction trial-block, *P < .05 vs. immediate group at same point in testing.

The general procedures for conditioning, extinction training and extinction retrieval were as described above, with the exception that at the time of extinction training, one group had been fear conditioned 24 hour earlier (= ‘delayed’), whereas another group had completed conditioning 10 minutes earlier (= ‘immediate’), as previously described [23,25]. For a schematic of the experimental design, see Figure 1A.

### Effects of varying the interval between cues during extinction training

**Figure 2:**
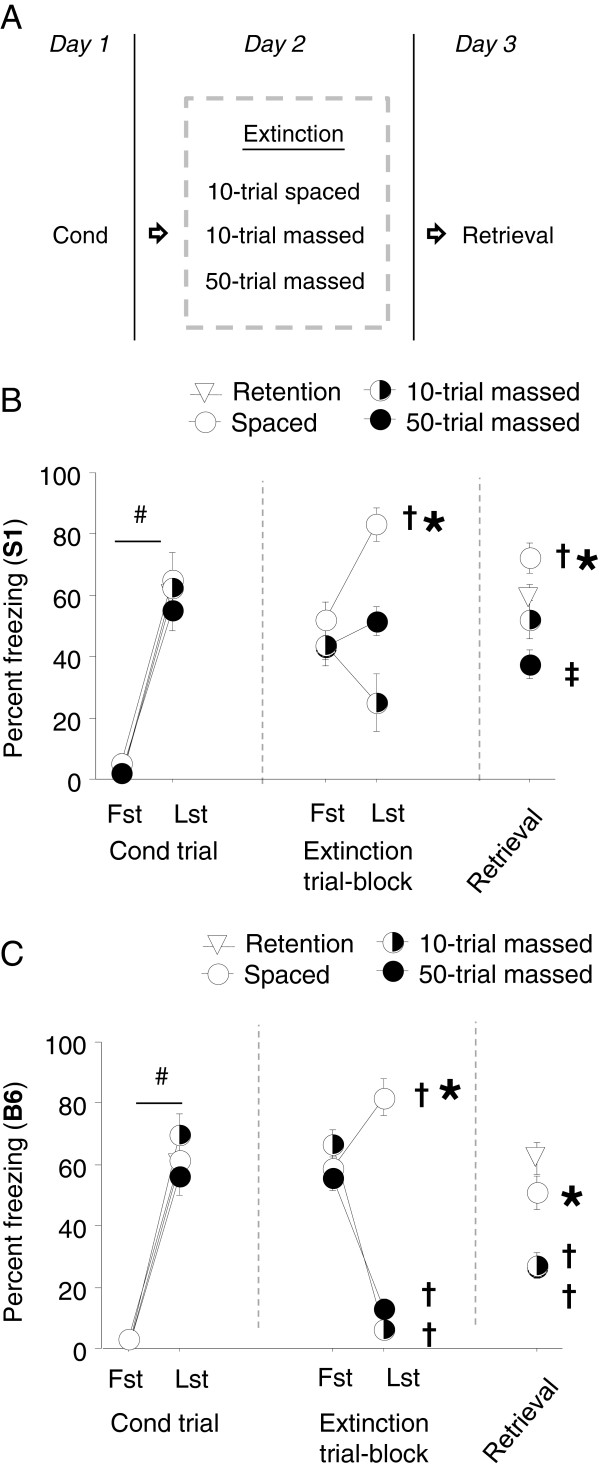
**Effects of spaced-CS extinction training. (A)** One day after conditioning, mice received 10-trial spaced (20 minute interval), 10-trial massed (5 second interval) or 50-trial massed extinction training. **(B)** In S1 mice, freezing significantly increased across extinction trial-blocks in the spaced group but did not change in the massed groups, such that freezing was significantly higher in the spaced group than the massed groups by the last trial-block. During extinction retrieval, freezing was significantly higher in the spaced group than the massed groups, but was not different from freezing in non-extinguished retention controls. The spaced group showed a significant increase in freezing on extinction retrieval relative to the first-trial block of extinction training (n = 8-26). **(C)** In B6 mice, freezing significantly decreased over extinction trial-blocks in the massed groups, but significantly increased in the spaced group, with higher freezing in the spaced group than in the massed groups by the final trial-block. On extinction retrieval, freezing was higher in the spaced group than the massed groups but no different from retention controls, and the massed groups had lower freezing than retention controls. The massed, but not spaced, groups showed significantly less freezing on retrieval than the first extinction training trial-block (n = 8-26). #*P* < .05 last (Lst) vs. first (Fst) conditioning trial, †*P* < .05 Lst extinction trial-block or retrieval vs. Fst extinction trial-block, *P < .05 vs. 10-trial massed group at same point in testing, ‡*P* < .05 vs. retention controls.

The general procedures for conditioning, extinction training and extinction retrieval were as described above, with the exception that during extinction training, one group received 10 x CS presentations each separated by 20 minutes (= ‘spaced’), whereas the other groups received either 10 × CS (= ‘10-trial massed’) or 50 × CS presentations (= ‘50-trial massed’) separated by 5 seconds, as previously described [26,27]. The massed group was exposed to the extinction training context for the equivalent duration as the spaced group (3 hours, 8 minutes, 30 seconds) to control for context exposure. An additional group of fear ‘retention controls’ received fear conditioning and no extinction training and were tested for fear on the extinction retrieval test for a comparison with the other groups. For a schematic of the experimental design, see Figure 2A.

### Effects of a single-CS exposure prior to extinction training

**Figure 3:**
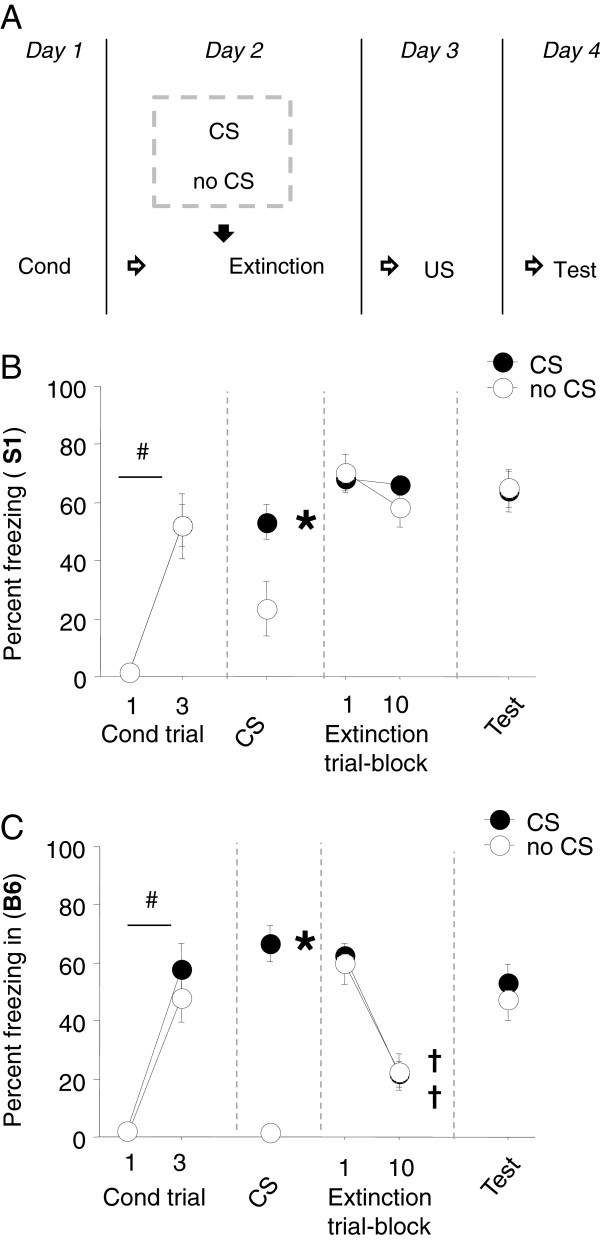
**Effects of single-CS exposure prior to extinction training. (A)** One day after conditioning, mice received either one CS presentation or no CS/context only 1 hour prior to extinction training. All mice then received equivalent extinction training. One day later, mice received unsignaled US presentations, and we then tested for fear the following day. **(B)** S1 mice significantly increased freezing across conditioning trials. During CS pre-exposure, freezing was higher during the 1xCS presentation than the no CS condition. Freezing did not change during across extinction trial-blocks regardless of CS pre-exposure. Freezing was also no different between groups during the post-reinstatement test (n = 10). **(C)** In B6 mice, freezing significantly increased across conditioning trials. During CS pre-exposure, freezing was higher to the CS presentation relative to the no CS condition. Freezing significantly decreased across extinction trial-blocks regardless of CS pre-exposure. Freezing did not differ between groups during the post-reinstatement test (n = 10). Data are means ± SEM. #*P* < .05 Lst vs. Fst conditioning trial, *P < .05 CS vs. no CS at same point in testing.

The general procedures for conditioning and extinction training were the same as above with the exception that 1 hour prior to extinction training, one group received a single (30-second) CS after a 3-minute acclimation period (= ‘1 × CS’), whereas another group was exposed to the context for the equivalent duration but received no CS presentation (= ‘0 × CS’). The 1 × CS group received 49 instead of 50 × CS presentations during extinction training to equate total CS presentations with the 0 × CS group. All phases of testing, including CS-pre-exposure, were conducted in Context A (i.e., the conditioning context) for consistency with previous CS-pre-exposure studies [29]. The day after extinction training, fear was reinstated via exposure to unsignaled footshock. After a 180-second baseline period, mice received 5 × USs (0.6 mA) over a 9.5 minute period (ITI = 6–150 seconds) and returned to the home cage 180 seconds after the final US. A fear probe test was conducted the following day via the typical extinction retrieval procedure. For a schematic of the experimental design, see Figure 3A.

### Extinction of a second-order conditioned cue

**Figure 4:**
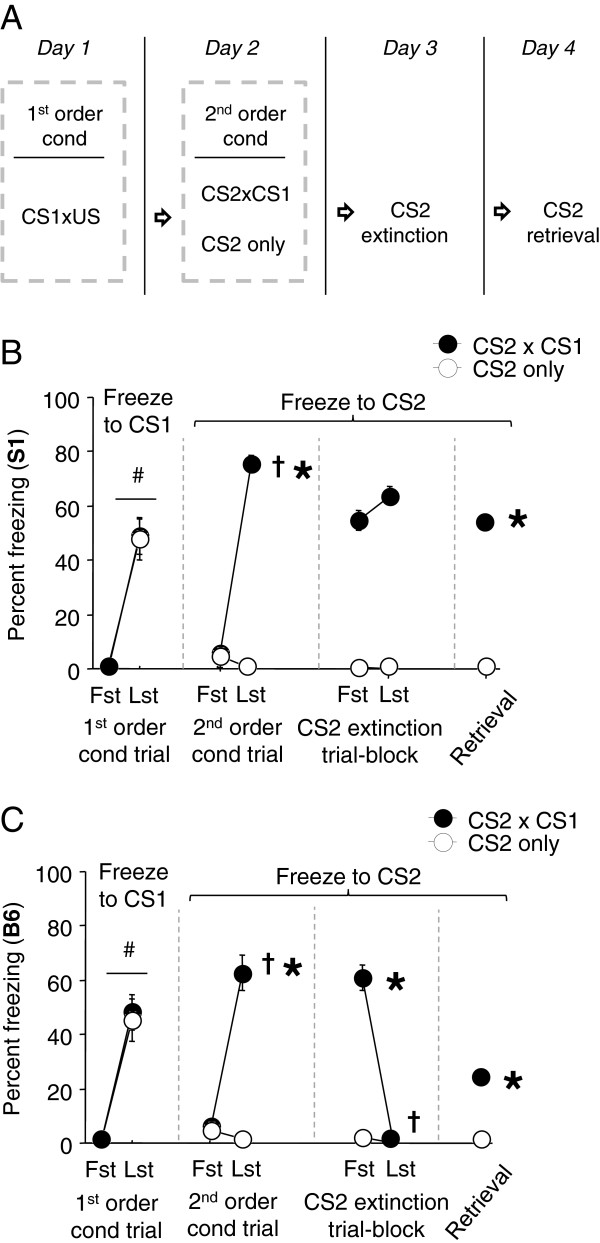
**Extinction of a second-order conditioned cue. (A)** All mice first received CS1xUS fear conditioning, 1 day prior to exposure to either CS2 paired with CS1 or CS2 exposure alone. All mice then received equivalent extinction training and retrieval testing. **(B).** S1 mice significantly increased freezing across CS1xUS pairings during conditioning. Freezing significantly increased across CS2 trials when the CS2 was paired with the CS1 and not when presented alone. Freezing to the CS2 did not change across extinction trial-blocks in either group and was significantly higher in the CS2-CS1 paired group than the CS2 only group during extinction retrieval (n = 5-10). **(C)**. B6 mice significantly increased freezing across CS1xUS pairings during conditioning. Freezing significantly increased across CS2 trials when the CS2 was paired with the CS1 and not when presented alone. Freezing to the CS2 significantly decreased across extinction trial-blocks in the CS2-CS1 paired group. Freezing was significantly higher in the CS2-CS1 paired group than the CS2 only group during extinction retrieval, but was lower relative to the first trial-block of extinction training (n = 5-10). #*P* < .05 last (Lst) vs. first (Fst) conditioning trial, †*P* < .05 Lst vs. Fst trial-block during 2nd order conditioning or extinction, *P < .05 vs. CS2-only at same point in testing.

The general procedures for conditioning, extinction training and extinction retrieval were as described above, with the following exceptions. One day after fear conditioning to a pure tone CS (CS1), one group received a second-order conditioning session in which a 75 dB white noise CS (CS2) was presented 5 × immediately prior to presentation of CS1 (without any concomitant US) (= ‘CS2×CS1’). Another group received 5 × CS2 presentations in the absence of CS1 during this phase (= ‘CS-only’). The following day, after a 120-second baseline, mice were given extinction training involving 16 × CS2 presentations. Fear to the CS2 was tested the following day via 2 × CS2 presentations. For a schematic of the experimental design, see Figure 4A.

### Statistical analysis

Data were analyzed using analysis of variance (ANOVA) with repeated measures for conditioning trial and extinction trial-block, followed by Newman Keuls *post hoc* tests where appropriate, or by paired t-tests. The threshold for statistical significance was *P* < .05.

## Results and discussion

### A short conditioning-extinction training interval reduces fear in S1 and impairs extinction in B6 mice

Previous work in rats has shown that reducing the typical one day interval between conditioning and extinction to less than one hour alters the efficacy of extinction training, as measured by fear levels during an extinction retrieval test. Some studies report a facilitatory effect of ‘immediate’ extinction [24], while others have observed impaired extinction [23,25,36]. We tested immediate extinction in the extinction impaired S1 and extinction intact B6 mouse strains using a 10 minute conditioning-extinction interval.

Results for S1 mice are shown in Figure 1B. Freezing was negligible at baseline and significantly increased across conditioning trials regardless of group assignment (F1,30 = 219.27, *P* < .01). During extinction training, baseline freezing trended higher (but not significantly) in the immediate group (as previously reported in rats, [23]), and was significantly lower during the first trial-block in the immediate than the delayed group, and then significantly increased across trial-blocks in the immediate but not delayed group (trial x group interaction: F1,30 = 14.13, *P* < .01, followed by *post hoc* tests). On the retrieval test, freezing was low at baseline and freezing to the CS was similar between the groups. In addition, in neither group was the level of freezing on retrieval different from that during the first-trial block of extinction training (i.e., prior to training).

Results for B6 mice are shown in Figure 1C. Freezing was negligible at baseline and significantly increased across conditioning trials regardless of group assignment (F1,18 = 277.44, *P* < .01). During extinction training, baseline freezing was not significantly higher in the immediate group. Freezing during the first trial-block was equivalent between groups and significantly decreased across trial-blocks during extinction training in the delayed but not immediate group, although there was a non-significant trend in the same direction in the immediate group (trial x group interaction: F1,18 = 8.59, *P* < .01, followed by *post hoc* tests). Moreover, on the extinction retrieval test freezing was similar between groups at baseline but significantly higher in the immediate than delayed group (t(18) = 2.26, *P* < .05). Both groups showed less freezing on retrieval relative to the first trial-block of extinction training (immediate: t(9) = 4.93, *P* < .01, delayed: (t(9) = 5.10, *P* < .01).

Taken together, these results provide a number of confirmatory and novel findings. First, under the typically employed testing procedure, in which there is a delay of one day between conditioning, S1 mice exhibited severely impaired extinction learning and retrieval. This is consistent with previous studies demonstrating an extinction deficient phenotype in this mouse strain, as compared to the B6 strain [15-19]. Prior work has also shown that this impairment is not an unalterable feature of these mice, but can be rescued by manipulations ranging from antidepressant drug treatment to deep brain simulation [17-19]. However, while studies in rats have found that conducting extinction training soon after conditioning can facilitate extinction [24], the current data found no evidence of a facilitatory effect of ‘immediate’ extinction in the S1 model. On the contrary, immediate extinction resulted in an extinction impairment in B6 mice, a finding in line with previous findings in rats [23,25,36].

Though immediate extinction did not affect extinction in S1 mice, initial fear levels prior to extinction training were reduced by this procedural manipulation. This could be indicative of a retardation in fear memory consolidation in the strain, such that one hour after conditioning, the memory cannot be fully retrieved. The underlying causes of this deficit are unclear, but could relate to abnormalities in molecular mechanisms underlying the early, protein and RNA synthesis-independent, phase of fear memory formation [37]. This raises interesting questions for future studies on the S1 strain.

### Spacing extinction cues increases fear in S1 mice and impairs extinction in B6 mice

Conditioned fear responses can be increased by interposing 1–2 month intervals between conditioning and test [38]. In addition, introducing long (20-minute) intervals between CS presentations during extinction training has been found to impair extinction in the B6 mouse strain [26,27] (c.f. facilitating effect of varying intervals [28]). We tested the effects of long (20-minute) cue spacing on extinction in S1, and for comparison with previous data, the B6 strain.

Results for S1 mice are shown in Figure 2B. Freezing significantly increased over conditioning trials (F1,39 = 120.10, *P* < .01) irrespective of group assignment. During extinction training, the spaced group significantly increased freezing over trial-blocks, whereas there was no significant change in the massed groups (trial-block x group interaction: F2,39 = 6.69, *P* < .01, followed by *post hoc* tests). The spaced group had significantly higher freezing than the massed group by the last trial-block. On the extinction retrieval test, the spaced group showed higher freezing than the massed group, but not fear retention controls that received no extinction training (F3,50 = 7.56, *P* < .01, followed by *post hoc* tests). Comparison of freezing during retrieval with the first-trial block of extinction training indicated significantly higher freezing across the test phases in the spaced group (t(7) = 3.20, *P* < .05), but neither massed groups. There was minimal baseline freezing across testing phases (Table 1).

**Table 1:** Levels of baseline (pre-CS) percent freezing across testing phases

		**S1**			**B6**	
***Varying conditioning-extinction training interval***						
	Cond	Extinction	Retrieval	Cond	Extinction	Retrieval
Immediate	0.0 ±0.0	15.1 ±22.0	10.2 ±15.7	0.0 ±0.0	16.1 ±23.3	5.0 ±3.1
Delayed	0.0 ±0.0	6.4 ±10.3	10.9 ±13.5	0.0 ±0.0	2.8 ±3.9	2.2 ±3.2
***Varying interval between extinction cues (3-shock cond)***						
	Cond	Extinction	Retrieval	Cond	Extinction	Retrieval
Spaced	0.0 ±0.0	0.7 ±1.3	2.1 ±2.0	0.2 ±0.8	4.7 ±3.5	3.0 ±2.6
10-trial massed	0.0 ±0.0	4.6 ±3.2	2.6 ±1.9	0.4 ±1.1	5.7 ±3.6	3.2 ±3.8
50-trial massed	0.0 ±0.0	1.7 ±2.5	3.5 ±3.6	0.0 ±0.0	2.4 ±2.3	1.7 ±2.5
***Varying interval between extinction cues (1-shock cond)***						
	Cond	Extinction	Retrieval	Cond	Extinction	Retrieval
Spaced	0.0 ±0.0	1.2 ±2.9	3.0 ±3.6	0.0 ±0.0	2.3 ±3.3	3.0 ±2.6
Massed	0.0 ±0.0	0.5 ±1.2	2.0 ±2.4	0.0 ±0.0	4.4 ±3.0	1.2 ±3.2
***Single-CS exposure prior to extinction training***						
	Cond	Extinction	Test	Cond	Extinction	Test
CS	0.0 ±0.0	9.2 ±9.1	29.4 ±18.9	0.0 ±0.0	11.1 ±17.5	33.9 ±22.9
No CS	0.0 ±0.0	19.4 ±18.2	35.6 ±19.9	0.0 ±0.0	5.6 ±13.9	18.6 ±16.9
***Extinction of a second-order conditioned cue***						
	Cond	Extinction	Retrieval	2nd Cond	Extinction	Retrieval
CS2 x CS1	0.4 ±0.7	0.0 ±0.0	0.0 ±0.0	0.1 ±0.4	0.0 ±0.0	0.0 ±0.0
CS2 only	0.4 ±0.9	0.0 ±0.0	0.0 ±0.0	0.0 ±0.0	0.0 ±0.0	0.0 ±0.0

Results for B6 mice are shown in Figure 2C. Freezing significantly increased across conditioning trials regardless of group assignment (F1,44 = 171.46, *P* < .01). During extinction training, freezing significantly decreased over trial-blocks in both massed groups, but significantly increased in the spaced group, such that freezing was significantly higher in the spaced group than the massed groups by the final trial-block (group x trial interaction: F2,44 = 31.60, *P* < .01, followed by *post hoc* tests). Group differences carried over to the extinction retrieval test, where the spaced group froze significantly more than the 10-trial and 50-trial massed groups, but not more than retention controls (F3,56 = 14.76, *P* < .01, followed by *post hoc* tests). Finally, the 10-trial massed (t(7) = 6.14, *P* < .01), 50-trial massed (t(25) = 5.71, *P* < .01), but not the spaced, group showed significantly lesser freezing during extinction retrieval than the first trial-block of extinction training. There was minimal baseline freezing on any testing phase (Table 1).

Consistent with previous findings [26,27], 10 x ‘spacing’ cue presentation during extinction training augmented fear over training trials in S1 and B6 mice. Freezing was also elevated during extinction retrieval after spaced training relative to mice that had received either 10 or 50 massed extinction trials, but not relative to mice that had received no extinction training at all. In B6 mice, these group differences on retrieval reflect impaired extinction after spaced training (contrasted with the intact extinction after massing), but not a long-term increase in fear over levels seen with no extinction at all. The retrieval differences in S1 mice reflect a similar pattern of impaired extinction, but no long-term fear increases. However, an interesting footnote to these data was the finding that S1 mice given 50 massed extinction trials showed lower freezing on retrieval than non-extinguished controls, which either suggests some, albeit marginal, extinction in this strain, or an augmentation of fear in the interval between conditioning and retrieval of the kind seen with much longer intervals in rats [38].

It should be noted that the design of these experiments does not exclude the possibility that increased freezing at the end of the long extinction training session (> 3 hours) was an artifact of very low levels of exploration, and even sleeping, erroneously detected as freezing. While low exploration could not explain the heightened levels of freezing on the extinction retrieval test, which was the equivalent length in the spaced and massed groups, it is possible that the long spaced training procedure impaired retrieval due to a lack of attention to the later cue presentations during extinction training. To address this caveat, we repeated the spacing procedure in another cohort of B6 mice that was conditioned with a single CS-US pairing. The rationale for this experiment is based on the theory that while a stronger conditioned fear response should be more prone to spacing-induced fear increases, weaker conditioning should be less prone [39].

The results of this experiment showed that B6 mice given either 10 spaced or 10 massed extinction trials showed equivalent freezing on conditioning (trial 1 spaced = 3.3 ± 2.4, trial 1 massed = 6.7 ± 4.2, n = 6-12), extinction training (first trial-block spaced = 50.0 ± 5.0%, first trial-block massed = 52.8 ± 10.0, last trial-block spaced = 27.8 ± 8.8, last massed = 16.8 ± 7.5, effect of trial-block: F1,16 = 16.50, *P* < .01) and extinction retrieval (spaced = 27.3 ± 5.8, massed = 43.6 ± 6.0). There was minimal baseline freezing across testing phases (Table 1). Thus, B6 mice showed intact fear extinction and no fear increases with spaced training after one-trial conditioning. We repeated the same procedure in S1 mice. Here, freezing on conditioning (trial 1 spaced = 1.3 ± 1.3, trial 1 massed = 0.0 ± 0.0, n = 6-16) was no different between groups, but the spaced, and not massed, group showed increased freezing across extinction trial-blocks during extinction training (first trial-block spaced = 46.8 ± 3.8%, first trial-block massed = 36.0 ± 6.7, last trial-block spaced = 69.8 ± 10.0, last massed = 36.0 ± 11.7, effect of spacing: F1,20 = 6.03, *P* < .05). Freezing on retrieval was not different between groups (spaced = 48.7 ± 4.4, massed = 44.4 ± 5.9). Again, there was minimal baseline freezing across testing phases (Table 1). These data indicate that, in contrast to B6 mice, fear increases with spacing in the S1 strain occurs even after one-trial conditioning.

These data make three important points. First, they discount the possibility that increased freezing in the spaced group after 3 × CS-US conditioning is an artifact of low exploration because the extinction training session duration is the same after 1 × CS-US experiment, and no increase in freezing is evident with this procedure. Second, and by extension, these data demonstrate that increased fear with spaced extinction training is determined in part by the intensity of the initial conditioning. This is consistent with the notion that the outcome of extinction training will be dependent upon the strength of the excitatory fear memory [40], and also agrees with the predictions of certain conceptual models of excessive fear in humans [39]. Third, the finding that S1 mice showed elevated fear even with one-trial conditioning suggests extinction-impaired populations may be more liable to this effect. This is consistent with the recent finding that extinction-impaired CB1 knockout mice displayed fear augmentation with massed training after being conditioned to a high intensity (0.8 mA) US, but not standard (0.6 mA) US [41].

The precise nature of the fear increases seen with spacing is unclear. Some authors have proposed that under some circumstances, the conditioned fear response to the CS can itself serve as a reinforcer to maintain fear and counter the effects of the CS-no-US association formed during extinction [39]. Alternatively, Cain and colleagues have suggested that spacing the presentation of CSs could promote fear by favoring the rehearsal of the original CS-US association with each presentation [26]. In a similar vein, it has been shown that there is more fear reconsolidation when conditioned fear stimuli are more widely spaced [42]. Our finding that spacing-induced fear increases, at least in B6 mice, was dependent upon the strength of conditioning would generally concur with these latter interpretations, assuming that stronger fear favored rehearsal/ reconsolidation over weaker conditioning. Aside from these interpretative questions, to our knowledge there is still no clear evidence of fear elevations with spaced extinction training in humans, and until such evidence is obtained, the relevance of these data in mice to clinical contexts should be viewed with caution.

### A single pre-extinction cue presentation is insufficient to prevent fear reinstatement

Presenting a conditioned cue one hour prior to extinction training has been found to facilitate extinction memory formation in humans [30,43], rats [29] (but see, [31]) and the B6 mouse strain [32,44], possibly by reactivating the fear memory to render it more labile and thereby sensitive to extinction training. Here we tested whether impaired extinction in the S1 strain could be rescued by pre-extinction cue exposure. B6 mice were also tested to ascertain the efficacy of this manipulation in a reference population.

Results for S1 mice are shown in Figure 3B. Freezing was negligible at baseline and significantly increased in freezing across conditioning trials regardless of group assignment (F1,18 = 59.64, *P* < .01). Baseline freezing prior to single CS presentation was slightly but not significantly elevated in the no CS group (Table 1). Cue presentation prior to extinction training elicited significant freezing, as compared to no-CS/context only exposure (t(18) = 2.70, *P* < .05). During extinction training, freezing did not significantly differ across trial-blocks, regardless of pre-training CS presentation. Following US-reinstatement, freezing was similar between groups during baseline, as well as during CS presentation, on the fear probe test. It was notable that baseline freezing on the fear probe test was higher than in other experiments (Table 1), reflecting contextual fear acquired during reinstatement (conducted in the same context). Strong contextual fear could potentially have affected (e.g., summed with) fear to the CS during the fear probe test and mitigated possibly fear-reducing effect of the pre-extinction CS exposure. This issue could be explored further by using a design involving multiple contexts.

Results for B6 mice are shown in Figure 3C. Freezing was negligible at baseline and significantly increased across conditioning trials irrespective of group assignment (F1,18 = 81.12, *P* < .01). Baseline freezing prior to the single CS trended higher in the CS group (Table 1). Freezing was elicited by CS presentation, and significantly more than no-CS/context only exposure (t(18) = 10.30, *P* < .01). Both the CS and no-CS groups showed a significant, and equivalent, decrease in freezing across extinction trial-blocks (F1,18 = 63.59, *P* < .01). Finally, freezing on the fear probe test after US-induced reinstatement was not different between the CS and no CS groups. As in S1 mice, baseline freezing was relatively high on this test and slightly higher in the CS than US group.

In contrast to recent findings in humans, rats and B6 mice [29,30,32,43,44] we did not find that exposure to the CS before extinction training facilitated extinction memory formation, either in B6 or S1 mice. One reason for this apparent discrepancy may be that we tested for extinction facilitation after unsignaled-US reinstatement, which is a strong fear-promoting procedure relative to other probes for facilitation such as spontaneous recovery and context-driven renewal. In other words, an extinction facilitation effect of CS presentation may have been overcome by such strong fear reinstatement. On the other hand, Monfils and colleagues were able to see a significant effect of pre-extinction CS presentation, in rats, under conditions of reinstatement [29]. Thus, there appear to be additional factors involved in determining the effects of CS presentation. Indeed, this is borne by a recent study in rats which found that pre-extinction CS exposure can actually *increase* fear following reinstatement (or renewal) [31]. Clearly, further studies will be necessary to elucidate the critical variables modulating this effect (for further discussion, see [45]).

### Impaired extinction of a second-order conditioned cue in S1 mice

Some authors have posited that anxiety disorders such as PTSD can be maintained by a process of second-order conditioning, in which previously neutral stimuli acquire associative strength after pairing with trauma reminders and then act as additional stimuli maintaining fear [46]. It is not known, however, whether individuals or populations differ in the ability to acquire and extinguish second-order conditioned fear memories. We therefore tested for this in the S1 and B6 mouse strains, using procedures previously described in rats [33,34].

Results for S1 mice are shown in Figure 4B. During conditioning to CS1, there was a significant increase in freezing across trials regardless of group assignment (F1,13 = 76.29, *P* < .01). On the second-order conditioning sessions, freezing to CS2 increased in the CS2-CS1 paired group across trials, but was negligible in the group presented with the CS2 alone (group x trial interaction: F1,13 = 163.23, *P* < .01, followed by *post hoc* tests). During extinction training, freezing to CS2 was significantly higher in the CS2-CS1 than CS2-only group (F1,13 = 203.55, *P* < .01), and did not change across trials in either group. Freezing was significantly higher to CS2 in the CS2-CS1 group than the CS2-only group on the extinction retrieval test (t(13) = 18.61, *P* < .01). There was minimal baseline freezing across testing phases (Table 1).

Results for B6 mice are shown in Figure 4C. Freezing significantly increased across CS1 conditioning trials regardless of group assignment (F1,13 = 74.76, *P* < .01). With second-order conditioning, freezing to CS2 increased across trials in the CS2-CS1 group but not the CS2-only group (group x trial interaction: F1,13 = 30.02, *P* < .01, followed by *post hoc* tests). During extinction training, freezing significantly decreased across trials in the CS2-CS1 group, whereas there was virtually no freezing in the CS2-only group (group x trial interaction: F1,13 = 72.53, *P* < .01, followed by *post hoc* tests). Freezing was significantly higher to CS2 in the CS2-CS1 than CS2-only group on the extinction retrieval test (t(13) = 10.62, *P* < .01). There was minimal baseline freezing across testing phases (Table 1).

These data show that both the S1 and B6 strains exhibit robust second-order fear conditioning under a procedure similar to those previously reported in rats [34]. This demonstrates that the CS1 served as a sufficient US (‘conditioned exciter’) to condition a new association with the CS2 that had no direct connection with footshock [33]. The conditioned response to CS2 successfully underwent extinction in B6 mice but, remarkably, could not be extinguished in S1 mice. As far as we are aware, this is some of the first evidence that an extinction-impaired population that also has a major deficit in extinguishing fear to stimuli only indirectly associated with the initial conditioning event. From a clinical perspective, these results suggest that post-trauma anxiety in certain at-risk individuals may be maintained by impaired extinction of second-order conditioned stimuli, and that it may be important to extinguish these stimuli in order to produce a fully effective therapeutic effect of extinction-based procedures such as exposure therapy. As such, it will be of great interest to elucidate the neurobiological basis of this form of extinction, and to determine to what degree it shares or differs from extinction of first-order conditioned stimuli. Studies are planned to investigate this further.

Further work is also needed to establish what abnormal learning processes underlie failed extinction of second-order conditioned stimuli. In one scheme, conditioning to CS1 might render S1 mice more attentive to the CS2-CS1 pairing during second-order conditioning, resulting in stronger learning of a CS2-CS1 association and subsequently poor extinction. One way to probe over-attention to the CS2 could be to test S1 mice for a deficit in the ability of prior conditioning to one CS to block conditioning to a second CS that is paired with the same US [47,48]. Alternatively, the S1 strain may have had a deficit in learning the conditioned inhibitor properties of the CS2 - i.e., learning that this stimulus is associated with the absence of an aversive outcome. This could also be more formally tested, for example by demonstrating that the presentation of the CS2 in the presence of the CS1 would inhibit fear to CS1 in B6 mice, but fail to do so in S1 mice. In lieu of such an analysis, however, a prior study found that, in contrast to B6, S1 mice fail to learn to reduce fear to a CS that had previously been explicitly unpaired with US [18] – consistent with a disruption of conditioned inhibition. This suggests that the S1 strain may have a fundamental problem with learning to suppress fear that manifests under various conditions, that includes but is not limited to the severe deficit in fear extinction that was initially found to characterize these mice [15,19].

## Conclusions

The current study examined the effects of manipulating the interval between conditioning and extinction training, the interval between CS presentations during training and exposure to a CS prior to training on fear extinction. It also examined extinction of a second-order conditioned fear response. Results provide further evidence that fear extinction is an ostensibly simple behavioral assay that is strongly influenced by multiple procedural variables and, importantly, is so in a strain-dependent manner. These findings in mice could have important implications for the application of extinction-based behavioral interventions, such as exposure therapy, for trauma-related anxiety disorders because it suggests that the efficacy of such interventions will be determined both by the procedural parameters employed and the clinical profile of the patient.

## Competing interests

The authors declare that they have no competing interests.

## Authors’ contributions

KM, NW, MC, and OG-C performed experiments. NW, NS and AH designed experiments. All authors read and approved the final manuscript.
